# Supplementary material for: Rational Photodeposition of Cobalt Phosphate on Flower-like ZnIn2S4 for Efficient Photocatalytic Hydrogen Evolution
Source: Molecules. 2024 Jan 17;29(2):465. doi: 10.3390/molecules29020465 (PMC10821521; doi:10.3390/molecules29020465)
Supplement: Supplementary file 1 [file molecules-29-00465-s001.zip › molecules-2824120-supplementary.pdf]

Supporting Information

Rational Photodeposition of Cobalt Phosphate on Flower-like $ZnIn_2S_4$ for Efficient Photocatalytic Hydrogen Evolution

Yonghui Wu [1], Zhipeng Wang [1], Yuqing Yan [1], Yu Wei [1], Jun Wang [1], Yunsheng Shen [1], Kai Yang [1], Bo Weng [2] and Kangqiang Lu [1,*]

[1] Jiangxi Provincial Key Laboratory of Functional Molecular Materials Chemistry, School of Chemistry and Chemical Engineering, Jiangxi University of Science and Technology, Ganzhou 341000, China; 13207070350@163.com (Y.W.); chemcatalyst@yeah.net (Z.W.); 18797912889@163.com (Y.Y.); 18225906201@163.com (Y.W.); wj15270473375@126.com (J.W.); shenyunsheng1@163.com (Y.S.); yangkai@jxust.edu.cn (K.Y.)

[2] cMACS, Department of Microbial and Molecular Systems, KU Leuven, 3001 Leuven, Belgium; bo.weng@kuleuven.be

* Correspondence: kqlu@jxust.edu.cn

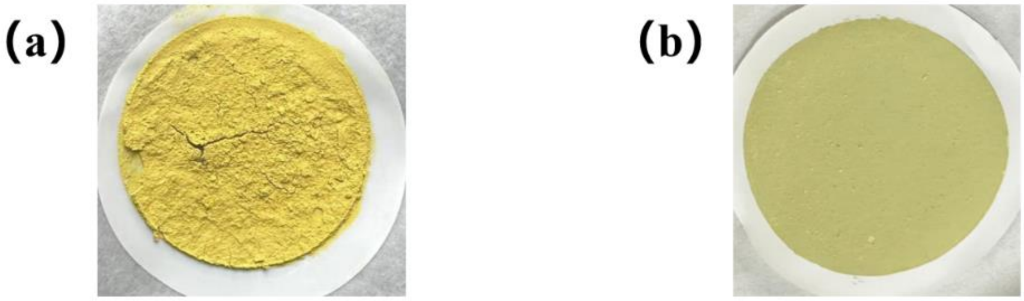

Figure S1. Schematic representation of the samples for $ZnIn_2S_4$ (**a**) and $ZnIn_2S_4$-5%Co-Pi (**b**)

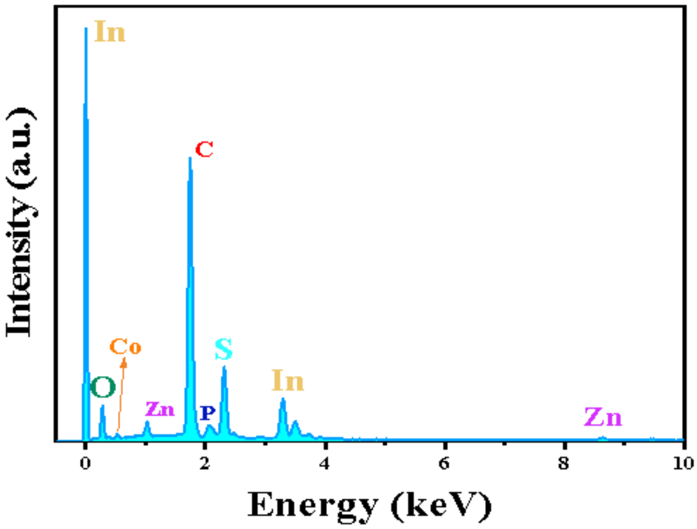

Figure S2. EDS spectrum of $ZnIn_2S_4$-5%Co-Pi

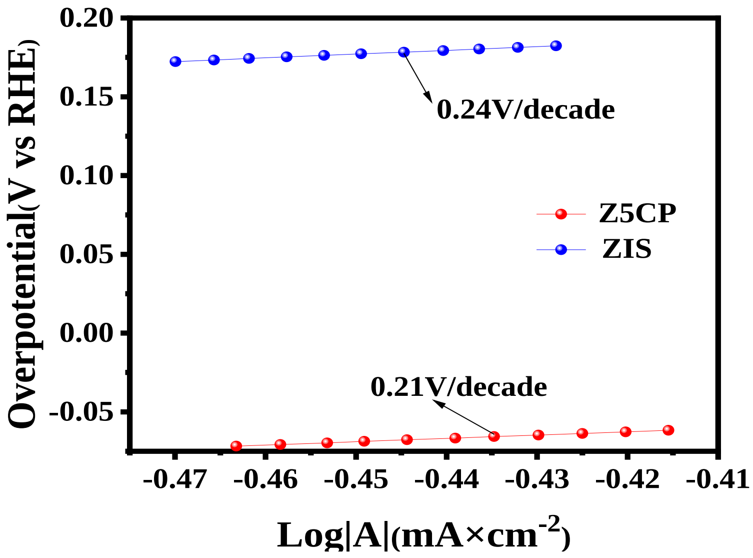

Figure S3. Tafel slope plots for $ZnIn_2S_4$ and $ZnIn_2S_4$-5%Co-Pi

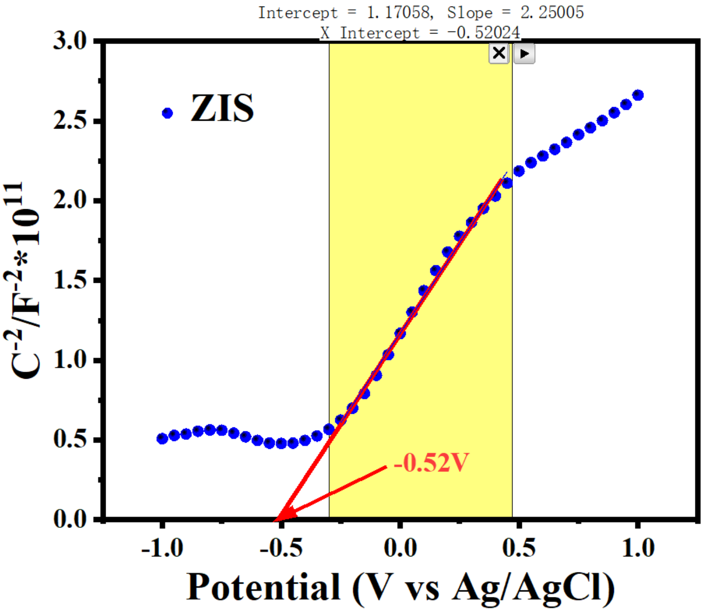

Figure S4. Mott-Schottky plots for $ZnIn_2S_4$.
